# Depth-dependent effects of crop rotation and monoculture on dissolved organic matter quantity and quality

**DOI:** 10.3389/fpls.2025.1668092

**Published:** 2025-08-21

**Authors:** Tianjing Ren, Guillaume Debaene, Aleksandra Ukalska-Jaruga, Bożena Smreczak

**Affiliations:** Department Soil Science and Environmental Analyses, Institute of Soil Science and Plant Cultivation-State Research Institute, Puławy, Poland

**Keywords:** dissolved organic matter, rotation, monoculture, soil depths, spectroscopy

## Abstract

**Introduction:**

Soil dissolved organic matter (DOM) regulates nutrient cycling and carbon sequestration, yet how cropping systems (rotation vs. monoculture) shape the vertical distribution and molecular traits of DOM remains unclear.

**Methods:**

We leveraged a long-term experiment (est. 1994) at the IUNG-PIB Agricultural Experimental Station, Osiny, eastern Poland, comparing a three-year rotation (winter oilseed rapewinter wheatspring barley) with continuous winter wheat. Soils were sampled at 030, 3060, and 6090 cm. Cold-waterextractable DOM was quantified as dissolved organic carbon (DOC), nitrogen (DON), and phosphorus (DOP). UVVis metrics (SUVA280​, E4/E6) characterized molecular features, and environmental drivers were identified via variable-importance analysis.

**Results and discussion:**

DOM concentrations declined with depth (P < 0.001). A management effect emerged only in the subsoil: DOC at 6090 cm was higher under monoculture than rotation (P < 0.05), indicating detectable but secondary cropping-system influences relative to depth controls. With depth, SUVA280​ increased and E4/E6 decreased consistently across systems, implying greater molecular weight and humification; thus, soil depth is the primary regulator of DOM molecular structure. Key drivers of DOM variability included soil organic carbon, total nitrogen, humus, available phosphorus, and depth. DOC variation was most associated with total nitrogen (14.92% contribution), total carbon (11.68%), and DOP (9.67%). DON was driven by DOC (17.64%), depth (16.00%), and available phosphorus (15.59%). DOP was shaped by humus (15.56%), total phosphorus (15.45%), and available phosphorus (15.43%). Collectively, these findings reveal pronounced depth-dependent differentiation of DOM quantity and traits in agricultural soils, with subsoil responses to management offering leverage points to optimize nutrient cycling and enhance long-term carbon storage.

## 1 Introduction

Soil dissolved organic matter (DOM), a vital component of terrestrial ecosystems, orchestrates biogeochemical cycles of carbon (C), nitrogen (N), and phosphorus (P) while governing soil fertility, microbial metabolism, and carbon sequestration dynamics. Comprising labile fractions such as dissolved organic carbon (DOC), nitrogen (DON), and phosphorus (DOP), DOM acts as both a substrate for microbial activity and a mobile vector for nutrient transport across soil profiles (Kalbitz et al., 2000; Smreczak and Ukalska-Jaruga, 2021). In agroecosystems, DOM dynamics are intricately linked to agricultural management practices, particularly crop crop rotation and monoculture, which alter root exudation patterns, residue inputs, and soil physicochemical properties (Chen et al., 2022; Smreczak and Ukalska-Jaruga, 2021; Undurraga et al., 2010). Despite its pivotal role in nutrient retention and carbon stabilization, the vertical stratification of DOM components and their molecular evolution across soil depths remain poorly resolved under contrasting cropping systems. This knowledge gap impedes the development of strategies to optimize soil health and climate resilience in intensively managed agricultural landscapes.

Current understanding of DOM dynamics has predominantly focused on surface soils (0–30 cm), where organic inputs are concentrated (Kalbitz et al., 2000; Rumpel and Kögel-Knabner, 2011). However, subsoil horizons (30–90 cm) represent critical yet underexplored reservoirs for stable organic carbon and nutrients, exhibiting distinct biogeochemical processes that diverge from surface layers. For instance, reduced microbial activity and slower organic matter turnover in subsoils may favor DOM stabilization or selective leaching of recalcitrant compounds (Spaccini et al., 2002). Crop management practices, such as monoculture and crop rotation, likely modulate these processes through divergent mechanisms: monoculture systems, characterized by uniform residue inputs and diminished biodiversity, may decrease DOM mineralization or accumulation of aromatic compounds (Zhang et al., 2025, 2021), whereas diversified crop rotations could enhance DOM complexity via heterogeneous organic inputs and rhizosphere interactions (Saadi et al., 2006). Yet, systematic assessments of DOM quantity, molecular traits, and their environmental drivers across soil depths in these systems are lacking, limiting predictive capacity for long-term soil carbon and nutrient cycling.

The molecular architecture of DOM—reflected in metrics such as aromaticity, molecular weight, and humification degree—serves as a fingerprint of its origin, stability, and ecological function (Ding et al., 2020; Wilson and Xenopoulos, 2008). Advanced spectroscopic techniques, including Ultraviolet-Visible absorbance (UV-Vis), enable non-destructive characterization of these properties, revealing shifts in DOM composition driven by microbial processing and environmental conditions (Marschner and Kalbitz, 2003). For example, increasing Specific Ultraviolet Absorbance at 280nm (SUVA_280_) and declining E4/E6 (absorbance at 465 nm divided by absorbance at 665 nm) ratios with depth suggest progressive humification and molecular weight amplification, processes that may be differentially regulated by cropping systems (Ukalska-Jaruga et al., 2021; Wang et al., 2015). However, disentangling the relative impacts of management practices versus inherent soil properties (e.g., organic carbon content, humus levels) on DOM dynamics requires robust analytical frameworks. Machine learning approaches, such as random forest modeling, offer unparalleled capacity to identify key drivers of DOM variability by quantifying the contributions of edaphic factors, management regimes, and depth-dependent interactions—a critical step toward predictive soil biogeochemistry (Ren et al., 2024).

This study bridges these knowledge gaps by investigating the vertical concentrations and quality distribution of DOM components (DOC, DON, DOP) across three soil depths (0–30, 30–60, and 60–90 cm) under crop rotation and monoculture systems. Employing cold-water extraction coupled with UV-Vis and Visible and Near-Infrared Spectroscopy (VIS-NIR) spectroscopy, we quantified DOM concentrations and quality, while random forest modeling elucidated the hierarchy of environmental drivers. Our objectives were to: (1) resolve depth-dependent variations in DOM quantity under contrasting cropping systems, (2) characterize shifts in DOM quality (e.g., aromaticity, humification) along soil profiles, and (3) identify key predictors governing DOM differentiation, including soil organic carbon, nutrient availability, and management practices. Based on existing knowledge gaps and ecological theories, we hypothesize that: (1) DOM concentrations decrease with soil depth, irrespective of cropping system, due to reduced organic inputs and microbial activity in deeper soil layers. (2) Crop crop rotation systems enhance DOM complexity and stability through increased diversity in organic matter inputs and microbial activity. Monoculture systems may reduce DOM mineralization, leading to the accumulation of recalcitrant DOM fractions, particularly at greater soil depths.

## 2 Materials and methods

### 2.1 Experimental design and soil sampling

The study was based on a long-term field experiment established in 1994 on an Haplic Luvisol (loamy sand) in Osiny Experimental Station (N: 51°28′, E: 22°30′) belonging to the Institute of Soil Science and Plant Cultivation, Pulawy, Poland (Feledyn-Szewczyk et al., 2024). For the purpose of this study soil samples were collected from different cropping systems: crop rotation and monoculture. The crop rotation system involves three-field crops: rapeseed, winter wheat, and spring barley (from 2005, spring wheat), whereas the monoculture system continuously cultivates winter wheat in the same plot every year (Siebielec et al., 2020). The size of each crop field in the rotation was 1 ha, which reflects the real crop production conditions (Martyniuk et al., 2016). The experiment was carried out with all crops cultivated at the same time, which made it possible to obtain full information from all fields in each year.

Soil sampling was performed in September 2022. Composite soil samples were collected from three depth intervals: 0–30 cm, 30–60 cm, and 60–90 cm, under both crop rotation and monoculture systems. At each depth, three subsamples were collected using a dedicated soil auger, then homogenized after removing visible plant residues. The samples were air-dried at room temperature and sieved through a 2-mm mesh prior to laboratory analysis. The basic physicochemical properties of the soils are presented in **Table 1**.

**Table 1:** Summary of soil physical and chemical properties under monoculture (M) and rotation (R) cropping systems across three soil depths (0–30 cm, 30–60 cm, and 60–90 cm).

Index	Descriptives	Monoculture_M			Rotation_R		
		0-30	30-60	60-90	0-30	30-60	60-90
clay (%)	Mean	16.33	20.33	28.83	14.83	22.50	32.50
	Median	17.00	18.00	29.00	15.00	22.50	33.00
	Variance	2.27	33.07	24.57	0.57	20.30	5.10
	Std. Deviation	1.51	5.75	4.96	0.75	4.51	2.26
	Minimum	14.00	17.00	20.00	14.00	15.00	30.00
	Maximum	18.00	32.00	34.00	16.00	29.00	36.00
sand (%)	Mean	71.83	69.67	60.83	73.67	67.17	56.00
	Median	72.00	71.50	60.00	74.00	67.50	55.50
	Variance	4.57	36.67	33.37	0.27	19.77	4.40
	Std. Deviation	2.14	6.06	5.78	0.52	4.45	2.10
	Minimum	69.00	58.00	55.00	73.00	60.00	54.00
	Maximum	75.00	75.00	71.00	74.00	73.00	59.00
silt (%)	Mean	26.00	24.83	25.00	24.83	25.33	27.33
	Median	26.00	25.50	26.50	25.00	25.50	27.50
	Variance	2.00	14.17	12.80	1.37	6.27	2.67
	Std. Deviation	1.41	3.76	3.58	1.17	2.50	1.63
	Minimum	24.00	18.00	20.00	23.00	22.00	25.00
	Maximum	28.00	29.00	28.00	26.00	28.00	29.00
moisture (%)	Mean	90.25	92.00	92.36	90.69	92.50	92.48
	Median	90.20	91.76	92.62	90.88	92.41	93.24
	Variance	0.71	1.23	1.27	0.19	1.21	5.96
	Std. Deviation	0.84	1.11	1.13	0.44	1.10	2.44
	Minimum	89.00	90.74	90.16	89.88	90.85	87.87
	Maximum	91.63	93.98	93.22	91.03	94.26	94.27
N_NH_4_ (mg/kg)	Mean	0.10	0.07	0.06	0.23	0.07	0.05
	Median	0.09	0.08	0.06	0.23	0.07	0.05
	Variance	0.00	0.00	0.00	0.01	0.00	0.00
	Std. Deviation	0.03	0.03	0.01	0.12	0.06	0.02
	Minimum	0.06	0.03	0.05	0.06	0.01	0.02
	Maximum	0.15	0.10	0.07	0.42	0.18	0.07
N_NO_3_ (mg/kg)	Mean	10.61	5.39	1.83	6.71	7.03	1.98
	Median	10.64	5.47	1.68	6.70	6.79	1.72
	Variance	1.82	7.21	0.92	8.84	1.77	1.09
	Std. Deviation	1.35	2.68	0.96	2.97	1.33	1.04
	Minimum	8.27	1.78	0.98	2.74	5.22	1.06
	Maximum	12.02	8.46	3.34	11.07	8.78	4.00
av_P (mg P_2_O_5_/100g)	Mean	13.33	5.97	0.56	12.41	2.88	0.71
	Median	13.13	5.18	0.64	11.87	2.92	0.64
	Variance	5.36	14.51	0.09	9.60	2.19	0.06
	Std. Deviation	2.32	3.81	0.30	3.10	1.48	0.25
	Minimum	10.74	1.07	0.17	9.06	1.32	0.43
	Maximum	16.95	11.23	0.87	16.05	5.48	1.03
CEC (cmol/kg)	Mean	4.93	5.45	8.04	3.34	5.03	9.05
	Median	4.72	5.06	8.25	3.28	4.99	9.08
	Variance	0.35	3.51	2.55	0.19	2.05	0.93
	Std. Deviation	0.59	1.87	1.60	0.44	1.43	0.96
	Minimum	4.37	3.94	5.08	2.94	2.74	7.60
	Maximum	5.77	9.10	9.53	4.14	7.19	10.44
pH_H_2_O	Mean	6.86	6.98	7.30	6.44	6.79	7.24
	Median	6.87	7.01	7.31	6.48	6.80	7.24
	Variance	0.00	0.01	0.00	0.02	0.02	0.02
	Std. Deviation	0.04	0.10	0.07	0.12	0.14	0.15
	Minimum	6.81	6.84	7.19	6.22	6.62	7.01
	Maximum	6.92	7.12	7.37	6.56	6.99	7.45
EC (µS/cm)	Mean	75.88	61.05	39.78	55.82	55.06	40.32
	Median	75.95	57.60	39.30	55.60	56.03	40.20
	Variance	55.31	90.50	3.05	25.85	12.02	29.94
	Std. Deviation	7.44	9.51	1.75	5.08	3.47	5.47
	Minimum	67.50	50.60	38.00	49.30	48.50	34.60
	Maximum	89.10	75.40	43.00	63.00	58.50	49.70
TOC (%)	Mean	0.82	0.42	0.22	0.56	0.23	0.16
	Median	0.81	0.31	0.22	0.57	0.22	0.16
	Variance	0.01	0.04	0.00	0.00	0.00	0.00
	Std. Deviation	0.12	0.19	0.02	0.05	0.04	0.03
	Minimum	0.69	0.28	0.19	0.46	0.20	0.13
	Maximum	0.99	0.68	0.26	0.61	0.28	0.20
Humus (%)	Mean	1.42	0.73	0.37	0.97	0.41	0.28
	Median	1.40	0.54	0.37	0.98	0.37	0.28
	Variance	0.04	0.11	0.00	0.01	0.00	0.00
	Std. Deviation	0.21	0.33	0.05	0.09	0.07	0.05
	Minimum	1.20	0.47	0.32	0.79	0.35	0.22
	Maximum	1.71	1.18	0.45	1.05	0.49	0.34
TN (%)	Mean	0.09	0.06	0.04	0.06	0.04	0.04
	Median	0.10	0.05	0.04	0.07	0.04	0.04
	Variance	0.00	0.00	0.00	0.00	0.00	0.00
	Std. Deviation	0.01	0.02	0.00	0.01	0.01	0.01
	Minimum	0.08	0.04	0.03	0.05	0.03	0.03
	Maximum	0.11	0.08	0.04	0.07	0.04	0.04
TC (%)	Mean	0.97	0.50	0.26	0.64	0.29	0.19
	Median	0.94	0.38	0.26	0.68	0.26	0.19
	Variance	0.02	0.05	0.00	0.00	0.00	0.00
	Std. Deviation	0.15	0.22	0.03	0.06	0.05	0.03
	Minimum	0.82	0.32	0.22	0.57	0.25	0.15
	Maximum	1.21	0.79	0.31	0.68	0.36	0.23
TP (mg/kg)	Mean	657.99	424.26	255.18	636.88	361.09	296.50
	Median	626.40	353.76	258.31	636.60	367.79	277.24
	Variance	9279.61	21373.86	5627.23	8426.05	5320.20	6163.90
	Std. Deviation	96.33	146.20	75.01	91.79	72.94	78.51
	Minimum	565.89	304.21	143.39	499.31	241.21	195.56
	Maximum	774.79	686.92	337.21	730.31	433.44	432.68

Values represent the mean of each variable, describing key soil texture (clay, sand, silt), moisture content, nutrient levels (N-NH_4_^+^, N-NO_3_-, available P), organic matter indicators (TOC, TN, TC, humus), pH, electrical conductivity (EC), cation exchange capacity (CEC), salinity, and total phosphorus (TP). Full descriptive statistics (mean, median, variance, standard deviation, minimum, and maximum) are available.

### 2.2 Determination of soil physicochemical properties

Soil pH was determined in a 1 mol·L^-^¹ KCl solution and water at a 1:2.5 (w/v) ratio using potentiometric method (PN-ISO 10390, 1997). Electrical conductivity (EC) was measured in the same 1:2.5 soil-to-water suspension using a conductivity meter (µS/cm) after equilibration. Soil particle size distribution was determined using the pressure hydrometer method according to PN-R-04032 (1998), which provided percentages of clay (<0.002 mm), silt (0.05–0.002 mm), and sand (2.0–0.05 mm). Total carbon (TC) and total nitrogen (TN) contents were quantified using a Vario Macro Cube elemental analyzer (Elementar Analysensysteme GmbH, Germany). Total organic carbon (TOC) was determined using the wet oxidation method with potassium dichromate (K_2_Cr_2_O_7_) and external heat application, followed by back-titration with ferrous ammonium sulfate FeSO_4_(NH_4_)_2_SO_4_·6H_2_O, according to PN-ISO 14235 (2003). Humus content was estimated using the equation: Humus = TOC × 1.724, where TOC represents the organic carbon content of the soil, assuming carbon constitutes 58% of humus. Available P were analysed using Egner-Rhiem method with calcium lactate at pH = 3,7 as extraction solution (Korzeniowska and Stanislawska-Glubiak, 2024). Cation exchange capacity (CEC) and hydrolytic acidity were determined using 1 mol L^-1^ ammonium acetate (pH 7.0) and calcium acetate (pH 8.2), respectively (Sumner and Miller, 1996). Total phosphorus (TP) was quantified after aqua regia digestion using inductively coupled plasma mass spectrometry (ICP-MS). Nitrate (NO_3_^-^) and ammonium (NH_4_^+^) ions were extracted with 1 mol L^-1^ potassium sulfide and analyzed colorimetrically (Keeney and Nelson, 1982).

### 2.3 DOM extraction

DOM was extracted using a cold-water extraction method. Five grams of air-dried and sieved soil (φ=2mm)were mixed with 50 mL of ultrapure water (Milli-Q, resistivity ≥ 18.2 MΩ·cm) at a 1:10 (w/w) ratio (Jones and Willett, 2006). The suspensions were shaken horizontally at 200 rpm for 24 hours at 25°C in the dark. After shaking, the mixtures were centrifuged at 3000 × g for 10 minutes, and the supernatants were filtered through 0.45μm polyethersulfone membrane filters (Millipore). The filtrates were collected and stored at –20°C prior to further analysis of DOM concentrations and spectral properties. The DOC and DON determined by a total organic carbon (TOC) and total nitrogen (TN) (multi N/C 2100 S, AJ, Germany) while DOP determined using ICP-MS apparatus.

### 2.4 UV-Vis and VIS-NIR spectroscopic analysis

UV-Vis spectroscopy analyzes DOM molecular composition and chemical properties (Williams et al., 2010; Yuan et al., 2018). Spectra are obtained using a UV-Vis spectrophotometer with 1 cm quartz cuvettes, using Milli-Q water as a reference. Specific UV absorbance (SUVA) is calculated as:


$$\text{SUVA}=\left(\text{UV}/\text{DOM concentration}\right)\times 100$$

SUVA280 (280 nm): indicates aromatic compound content (e.g., lignin, humic substances). Higher values reflect greater aromaticity. SUVA465 (465 nm): indicates the relative content of humic substances (e.g., fulvic and humic acids). E4/E6 ratio (absorbance at 465 nm divided by absorbance at 665 nm): serves as a proxy for DOM molecular size and degree of humification. Higher E4/E6 values are typically associated with low-molecular-weight, microbially derived, and more labile DOM fractions, whereas lower E4/E6 values suggest high-molecular-weight, highly condensed, and more humified DOM components (Ukalska-Jaruga et al., 2021).

The visible and near-infrared (VIS-NIR) measurements were carried out using a PSR-3500^®^ spectroradiometer (Spectral Evolution, Lawrence, MA, USA), operating in the 350–2500nm range (Ukalska-Jaruga et al., 2021). Reflectance values were converted to pseudo-absorbance (A^*^) using the transformation A^*^=-log_10_(R), as provided by the instrument software. The spectral region beyond 1870nm was excluded from analysis due to a marked increase in noise and poor signal-to-noise ratio, primarily caused by strong water absorption bands and reduced detector sensitivity. These effects are particularly pronounced when measuring aqueous extracts in diffuse reflectance mode using a cuvette, resulting in distorted or unstable pseudo-absorbance values that compromise the reliability of the data in this region. Before measurement, DOM solutions were equilibrated to room temperature (~20°C), gently homogenized, and placed in 10mm pathlength quartz cuvettes. Spectral data were collected in pseudo-absorbance mode using a tungsten-halogen light source, and the instrument was calibrated against a Milli-Q water blank before each batch.

### 2.5 Statistical analysis and modeling

All experimental data were tested for normality and homogeneity of variance prior to statistical analysis. Differences in DOM concentrations and spectral indices across cropping systems and soil depths were assessed using one-way ANOVA followed by Tukey’s *post hoc* test (*P* < 0.05). To explore the key environmental drivers of DOM dynamics, random forest (RF) modeling was performed using the “randomForest” package in R (version 4.3.1) (Ren et al., 2024). Predictor variables included soil physicochemical properties (e.g., TOC, TN, clay content, pH, humus) and categorical factors (cropping system and soil depth). Variable importance was assessed based on the percentage increase in mean squared error (% IncMSE). VIS-NIR spectra preprocessing was done with Unscrambler 10.3 (Camo Analytics, Oslo, Norway). It included Savitzky-Golay smoothing (11-point window, 2^nd^ order polynomial) followed by baseline offset correction. This procedure removed minor negative values caused by instrumental drift, without altering spectral features.

## 3 Results

### 3.1 Crop systems influence quantity of DOM components

**Figure 1:**
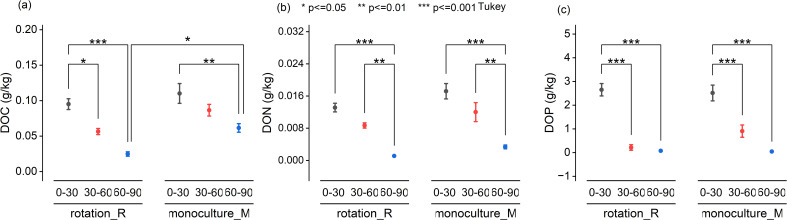
Vertical distribution of soil dissolved organic carbon (DOC), nitrogen (DON), and phosphorus (DOP) under rotation and monoculture systems across different soil depths.Concentrations of cold-water extractable dissolved organic carbon (DOC, panel **a**), dissolved organic nitrogen (DON, panel **b**), and dissolved organic phosphorus (DOP, panel **c**) across three soil depths (030 cm, 3060 cm, and 6090 cm) under rotation (R) and monoculture (M) cropping systems. Data are presented as mean ± standard error (n = 12). Significant differences among depths within each system were evaluated by Tukey's HSD test. Significance levels are indicated as: p < 0.05 (*), p < 0.01 (**), and p < 0.001 (***).

Across all three soil depths (0–30 cm, 30–60 cm, and 60–90 cm), concentrations of all DOM components—DOC, DON, and DOP—consistently decreased with increasing depth under both monoculture and crop rotation systems (**Figure 1**). A significant difference between cropping systems was observed only for DOC in the deepest soil layer (60–90 cm), where monoculture plots exhibited higher DOC concentrations than crop rotation plots (*P* < 0.05; **Figure 1a**). No significant differences in DON or DOP concentrations were found between cropping systems at any depth (**Figures 1b, c**). Statistical comparisons among soil layers revealed significant vertical stratification for DOC and DON (*P* < 0.05 across all depths). In contrast, DOP only differed significantly between the surface layer (0–30 cm) and the subsoil layers (30–60 and 60–90 cm), with no significant difference between the latter two, indicating a more stable vertical distribution. These patterns suggest that the topsoil plays a critical role in nutrient availability, particularly for phosphorus.

### 3.2 Crop systems influence the quality of DOM components

**Figure 2:**
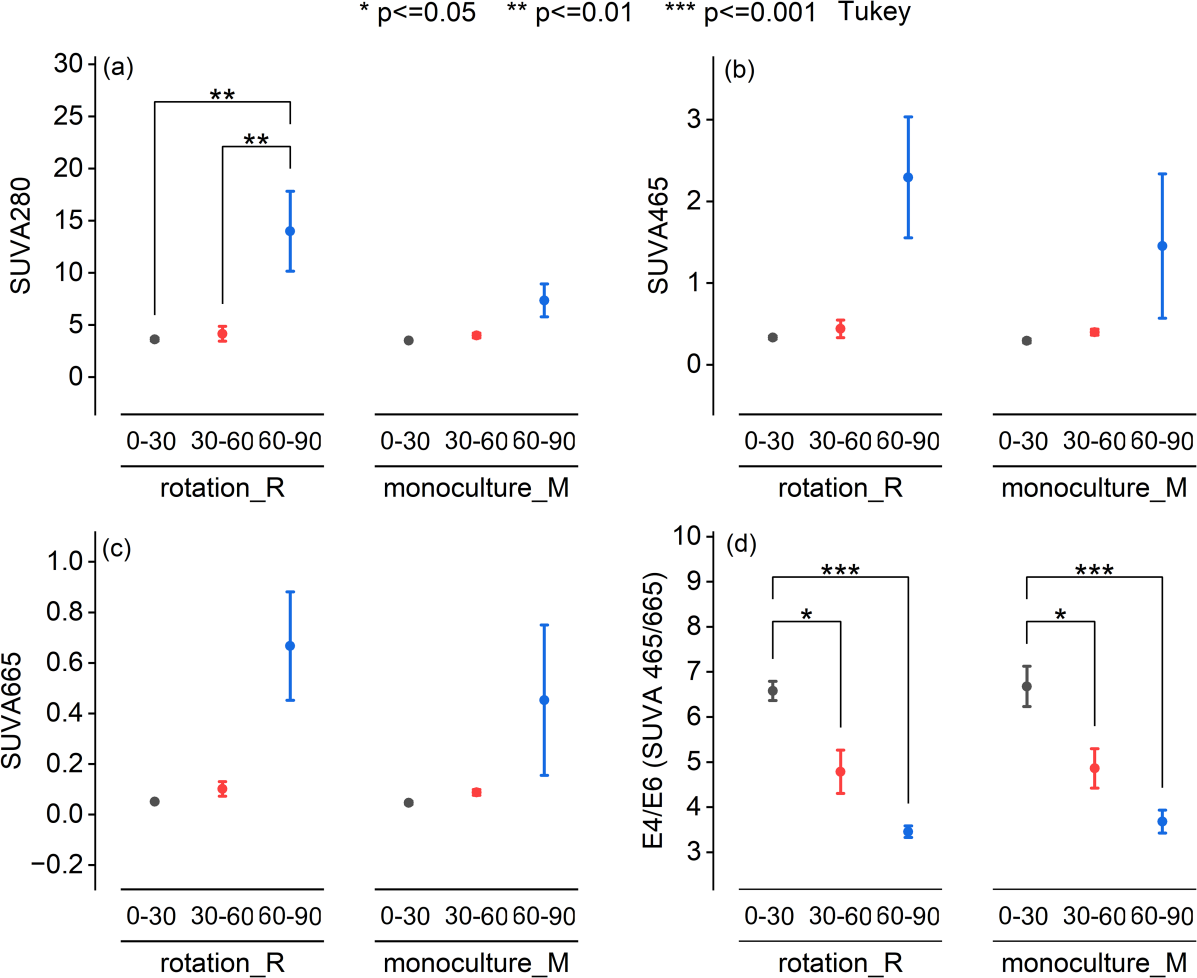
UV-Vis spectrum characteristics of cold-water extractable dissolved organic matter (DOM) across soil depths under rotation and monoculture systems. Spectral indices derived from UVVis absorbance of cold-water extractable DOM in soils sampled from three depths (030 cm, 3060 cm, and 6090 cm) under rotation (R) and monoculture (M) cropping systems. **(a)** SUVA280: Specific UV absorbance at 280 nm, indicating aromaticity and protein-like content. **(b)** SUVA465: Specific UV absorbance at 465 nm, associated with humic substances. **(c)** SUVA665: Specific UV absorbance at 665 nm, reflecting the presence of highly aromatic and condensed structures. **(d)** E4/E6 ratio (SUVA465/SUVA665), indicating molecular size and humification degree of DOM. Data are presented as mean ± standard error (n = 12). Significant differences among depths within each system were evaluated by Tukey's HSD test. Significance levels are indicated as: p < 0.05 (*), p < 0.01 (**), and p < 0.001 (***).

**Figure 3:**
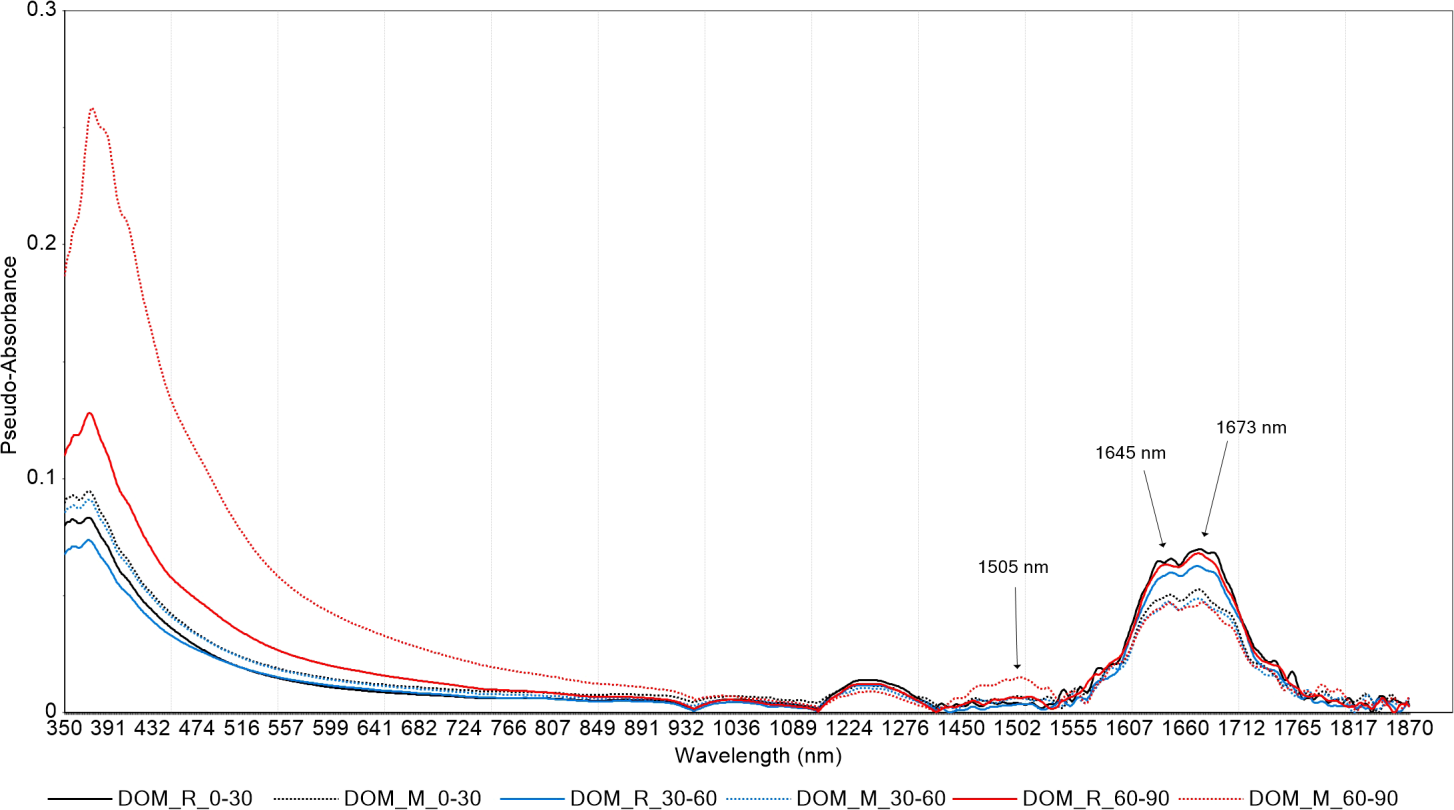
Mean VISNIR spectra of cold-water extractable dissolved organic matter (DOM) from soils under different cropping systems and depths. Average pseudo-absorbance spectra (3501850 nm) of cold-water extractable dissolved organic matter (DOM) under rotation (R) and monoculture (M) cropping systems across three soil depths (030 cm, 3060 cm, and 6090 cm). The spectral curves represent the mean pseudo-absorbance values from triplicate or composite samples. Solid lines indicate rotation (DOM _R) and dotted lines indicate monoculture (DOM _M). Colors denote soil depth: black (030 cm), blue (3060 cm), and red (6090 cm).

UV-Vis spectroscopic analysis revealed no significant differences in DOM quality between crop rotation and monoculture systems. Specific ultraviolet absorbance at 280 nm (SUVA280), a proxy for DOM aromaticity, showed a significant increase with depth in the crop rotation system (*P* < 0.01; **Figure 2a**), particularly in the 60–90 cm layer. While SUVA465 and SUVA665 also exhibited elevated values in the deepest soil layer under both systems, these differences were not statistically significant (**Figures 2b, c**). In contrast, the E4/E6 ratio, indicative of DOM molecular size and humification, declined significantly with depth in both systems (**Figure 2d**), confirming a trend toward more humified, high-molecular-weight DOM in subsoils. **Figure 3** presents the means of VIS-NIR spectra at the three depths for the two cropping systems. In the visible region, the monoculture system presented higher pseudo-absorbance. On the contrary in the NIR region, pseudo-absorbance was higher with the crop rotation system. At 60–90cm depth, VIS–NIR mean spectra revealed consistently higher absorbance values in monoculture compared to crop rotation. This trend aligns with UV–Vis and chemical data showing elevated DOC concentrations under monoculture, suggesting that VIS–NIR spectroscopy may capture DOC-related variation when concentration differences are sufficiently large. These patterns indicate that soil depth, more than cropping system, governs the molecular complexity and stability of DOM in agricultural soils.

### 3.3 Key predictors governing DOM differentiation

**Figure 4:**
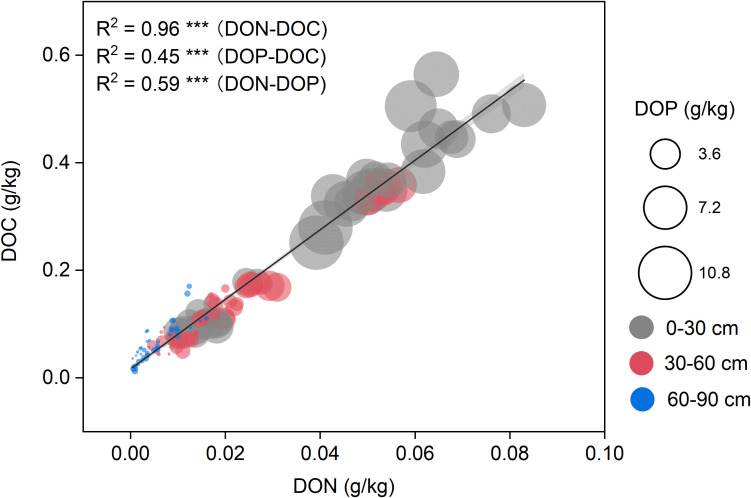
Relationships among dissolved organic carbon (DOC), dissolved organic nitrogen (DON), and dissolved organic phosphorus (DOP) across soil depths. Scatter plots illustrate the correlations between DOC and DON (R² = 0.96), DOP and DOC (R² = 0.45), and DOP and DON (R² = 0.59), all statistically significant (*p* < 0.001, ***). Points are color-coded by soil depth: grey (030 cm), red (3060 cm), and blue (6090 cm). Circle sizes are proportional to DOP concentrations, providing a third dimension to the bivariate relationship.

**Figure 5:**
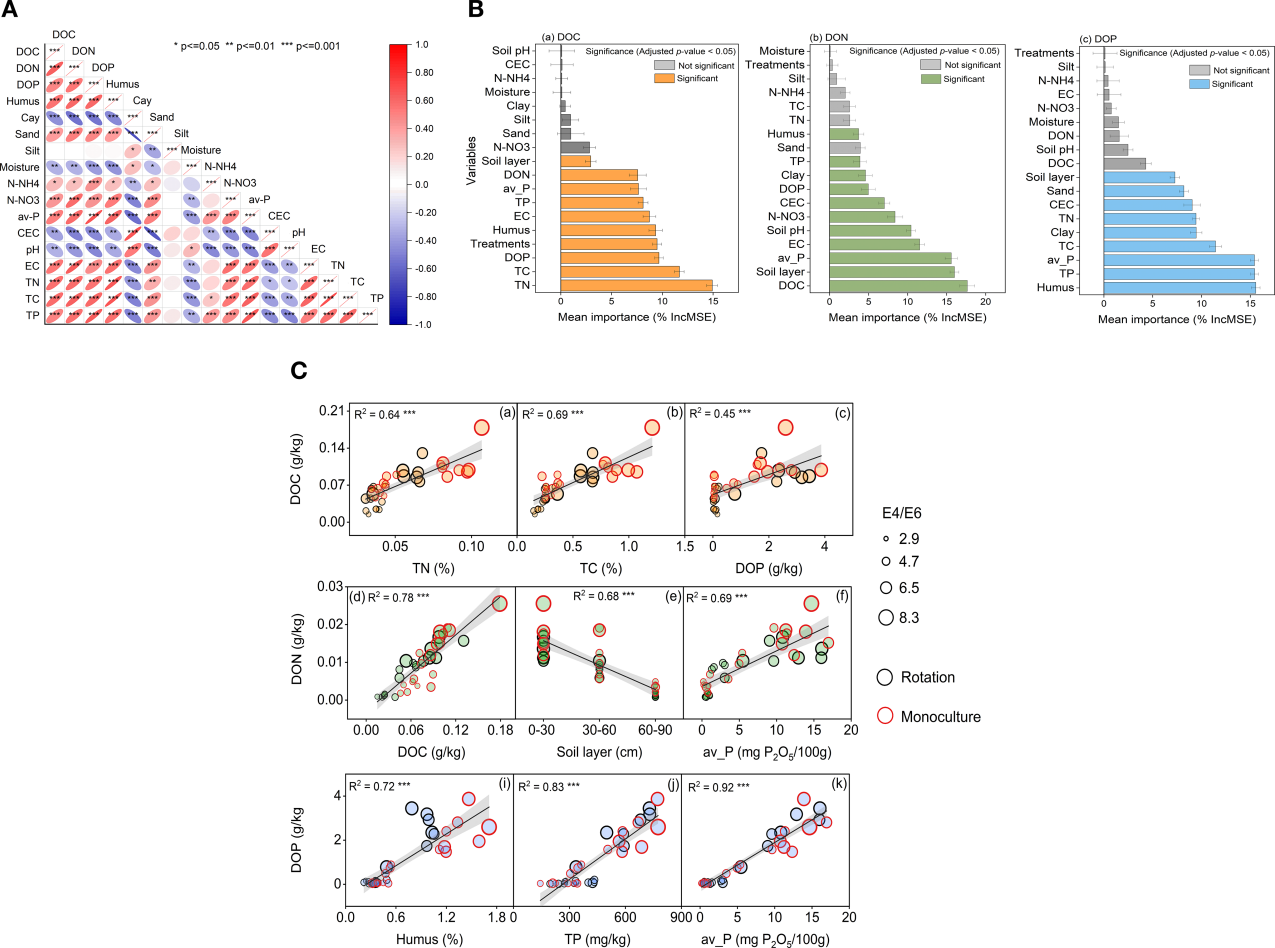
Integrated controls and relationships of cold-waterextractable DOM. **(A)** Spearman correlation matrix among CWE-DOC, CWE-DON, CWE-DOP and soil physicochemical variables; ellipse orientation and color encode r (red positive, blue negative), with significance marked as *P < 0.05, **P < 0.01, ***P < 0.001. **(B)** Random-forest variable importance (%IncMSE) for predicting DOC, DON and DOP; bars show mean ± SD; colored bars denote significant predictors (adjusted *P* < 0.05; orange = DOC, green = DON, blue = DOP). **(C)** Linear relationships between DOM components and key soil properties across cropping systems; points are rotation (black) and monoculture (red), point size scales with E4/E6 (aromaticity/humification proxy), shaded bands indicate 95% confidence intervals; all regressions *P* < 0.001.

Correlation analysis and random forest modeling identified key soil properties associated with DOM component variability (**Figures 4**, **5**). DOC variation was primarily driven by total nitrogen (IncMSE = 14.92%), total carbon (11.68%), and DOP (9.67%) (**Figure 5A**). For DON, the most influential predictors were DOC (17.64%), soil depth (16.00%), and available phosphorus (15.59%) (**Figure 5B**). DOP was most strongly influenced by humus content (15.56%), total phosphorus (15.45%), and available phosphorus (15.43%) (**Figure 5C**). Subsequent linear regression confirmed the robustness of these relationships, emphasizing the importance influence of both soil characteristics and management practices on the biogeochemical behavior of DOM in agricultural systems.

## 4 Discussion

### 4.1 Effects of cropping systems on DOM concentration

Our findings demonstrate that soil depth exerts a stronger control than cropping systems on the distribution of dissolved organic matter (DOM) in agricultural soils. Concentrations of dissolved organic carbon (DOC), nitrogen (DON), and phosphorus (DOP) consistently decreased with increasing depth across all layers (0–30 cm, 30–60 cm, and 60–90 cm). This pronounced vertical stratification is consistent with previous studies (Kaiser and Kalbitz, 2012; Kalbitz et al., 2000), and can largely be explained by reduced inputs of plant residues, lower root exudation, and diminished microbial activity in subsoils. The minimal differences in DOM concentrations between crop rotation and monoculture systems in the upper soil layers likely reflect long-term homogenization of soil properties due to decades of similar fertilization, tillage, and management practices. Although crop rotation generally enhances microbial diversity and activity through more diverse organic inputs and root exudates (Chen et al., 2022). In contrast, monoculture tends to reduce microbial diversity and promote microbial communities specialized in decomposing uniform crop residues (Zhang et al., 2025), these effects appear insufficient to cause measurable differences in DOM concentrations in surface soils. Significant cropping system effects were detected only in the deepest layer (60–90 cm), where monoculture soils exhibited higher DOC levels than rotation soils. This pattern likely reflects reduced microbial diversity and slower decomposition rates under monoculture, promoting the accumulation of more recalcitrant DOC fractions at depth (Findlay and Parr, 2017; Zhang et al., 2025). Furthermore, prolonged monoculture could impair soil aggregate stability, facilitating vertical transport and retention of particulate organic matter and associated DOC in deeper horizons (Dou et al., 2025; Rumpel and Kögel-Knabner, 2011; Xu et al., 2021). In contrast, DON and DOP concentrations did not differ significantly between cropping systems, implying these nutrient fractions are more strongly governed by intrinsic soil processes, such as microbial mineralization and adsorption interactions, rather than management practices (Helfenstein et al., 2018; Karl and Björkman, 2015). The significant variation of DOP primarily between surface (0–30 cm) and deeper soil layers (30–90 cm) is consistent with the established “surface enrichment–deep depletion” phosphorus distribution pattern (Jobbagy and Jackson, 2001), driven by the strong affinity of phosphate compounds for mineral adsorption sites.

### 4.2 Effects of cropping systems on DOM quality

Despite contrasting management practices, no significant differences in DOM molecular characteristics were detected between crop rotation and monoculture systems across all soil depths. Spectroscopic analyses consistently highlighted the dominant influence of soil depth over management effects in shaping DOM quality. UV–Vis results showed a marked increase in aromaticity (higher SUVA280; **Figure 2a**) and humification (lower E4/E6 ratio; **Figure 2d**) with depth, indicating progressive molecular condensation and stabilization of DOM in subsoils. Complementary VIS–NIR analyses provided additional compositional insights across the visible (400–700 nm) and near-infrared regions (1450–1750 nm) (**Figure 3**). Although overall DOM concentrations declined with depth, pseudo-absorbance in the visible region increased slightly in the deepest layer (60–90 cm), reflecting enhanced accumulation of aromatic, humified compounds with stronger visible light absorption. Near-infrared spectra revealed a broad absorption peak around 1505 nm, associated with labile DOM constituents such as carbohydrates and microbial metabolites (Stenberg et al., 2010; Workman and Weyer, 2007). This feature was particularly pronounced under monoculture in the deepest soil, suggesting preferential leaching and accumulation of microbially derived hydrophilic DOM fractions. In contrast, sharper peaks at 1645 nm and 1673 nm, linked to aromatic and phenolic structures (e.g., lignin derivatives) (Ben-Dor and Banin, 1995; Workman and Weyer, 2007), were less intense in monoculture at depth, implying reduced humification compared to rotation soils. Depth-driven changes in DOM composition likely result from selective microbial decomposition of surface-derived labile compounds, coupled with downward migration and preservation of recalcitrant aromatic fractions (Sanderman et al., 2009; Weishaar et al., 2003). Cropping systems may influence these processes indirectly through microbial activity. Enhanced enzyme production under crop rotation (e.g., oxidative enzymes such as phenol oxidase and peroxidases, or hydrolases like β-glucosidase) could accelerate DOM mineralization, thereby limiting the accumulation of recalcitrant aromatic fractions and contributing to lower DOC concentrations in rotation subsoils compared with monoculture systems (Saadi et al., 2006). Additionally, oxygen-limited conditions in subsoil layers may promote oxidative condensation reactions, producing structurally complex humic substances with enhanced stability and long-term carbon storage potential, albeit potentially limiting microbial bioavailability (Bravo-Escobar et al., 2024; Khan et al., 2024; Rumpel and Kögel-Knabner, 2011). Although root activity and organic inputs differ between cropping systems, UV–Vis indices were not sensitive enough to resolve subtle molecular differences, likely due to long-term homogenization of soil properties under similar fertilization regimes and the overriding effect of soil depth. Future research employing high-resolution molecular analyses (e.g., FT-ICR-MS, ^13^C-NMR) combined with microbial community profiling and enzymatic assays would allow a more detailed examination of DOM chemodiversity and stabilization pathways (Ding et al., 2020). Such integrative approaches could reveal nuanced management effects not detectable with conventional spectroscopic techniques, advancing understanding of DOM transformation mechanisms and their implications for soil carbon sequestration.

### 4.3 Key environmental controls and implications for subsoil carbon and nutrient management

Using correlation analysis and Random Forest modeling, this study systematically identified the primary environmental factors regulating DOM component distribution. The results reveal that distinct drivers govern different DOM fractions, reflecting their independent roles within biogeochemical cycles. For DOC, soil TC and TN emerged as dominant predictors, underscoring the pivotal role of soil organic matter as the primary source of dissolved carbon. This finding aligns with the well-established paradigm that DOM originates largely from the mineralization and leaching of soil organic matter pools (Bolan et al., 2011; Kaiser and Kalbitz, 2012; Karavanova, 2013). Additionally, cropping systems contribute to DOC dynamics by altering carbon input pathways (**Figure 5A**). For instance, the accumulation of DOC in deeper monoculture layers may reflect the selective preservation of recalcitrant aromatic compounds, as evidenced by the depth-related increase in SUVA280 (**Figure 2a**) and a higher visible region (400–700nm) at 60-90 cm by VIS-NIR (**Figure 3**). Linear regression analysis supports this relationship, suggesting that augmenting soil organic carbon (SOC) inputs—through crop residues, root turnover, or organic amendments—can effectively elevate DOC concentrations (Ren et al., 2024; Smreczak and Ukalska-Jaruga, 2021). In contrast, DON dynamics were primarily influenced by DOC (IncMSE = 17.64%), soil depth (IncMSE = 16.00%), and available phosphorus (IncMSE = 15.59%). The strong linkage between DOC and DON underscores the structural coupling of organic nitrogen within DOM (**Figure 3**), consistent with co-migration or co-mineralization mechanisms (Neff et al., 2003). The pronounced effect of soil depth reflects the vertical gradients in microbial activity, root distribution, and organic inputs, with DON typically declining with depth due to reduced biological turnover and substrate availability (Berman and Bronk, 2003; Sipler and Bronk, 2015). Intriguingly, available phosphorus emerged as a key predictor of DON, hinting at cross-regulation between phosphorus and nitrogen cycles. Under phosphorus-limited conditions, microorganisms may accelerate organic nitrogen mineralization to meet nutrient demands, thereby elevating DOP levels in the soil solution (Karl and Björkman, 2015). DOP, in turn, was predominantly controlled by humus content (IncMSE = 15.56%), total phosphorus (IncMSE = 15.45%), and available phosphorus (IncMSE = 15.43%). These factors collectively illustrate the interplay between organic and inorganic phosphorus pools and their modulation by microbial processes. Humus acts as a long-term organic phosphorus reservoir, gradually releasing DOP via microbial decomposition or enzymatic hydrolysis (Kalbitz et al., 2000; Zsolnay, 2003). The concurrent influence of total and available phosphorus suggests that DOP production hinges on the equilibrium between phosphorus storage and microbial demand (Karl and Björkman, 2015). Effective subsoil phosphorus management should thus balance the availability of labile phosphorus with the stabilization of organic phosphorus within the humus matrix. A notable asymmetry exists in the relationship between DOP and DOC: DOP significantly predicts DOC, but DOC does not reciprocally influence DOP. This likely reflects the structural and functional hierarchy within DOM. DOP compounds, such as phospholipids and nucleotides, contribute carbon to the DOC pool, with their release—particularly during microbial turnover—enriching both phosphorus and carbon in the dissolved phase (Karl and Björkman, 2015). Conversely, DOC encompasses a diverse array of carbon-containing compounds, most of which lack phosphorus (Ding et al., 2020), explaining why DOC fluctuations do not necessarily affect DOP. Moreover, DOP release is governed by phosphorus-specific processes, such as microbial phosphorus demand and the mineralization of humus-bound phosphorus, which operate independently of broader DOC dynamics (Richardson and Simpson, 2011). From an agricultural management perspective, these insights emphasize the importance of enhancing SOC storage and optimizing nutrient supply to regulate DOM dynamics, particularly for carbon, nitrogen, and phosphorus retention and slow release in subsoils.

## 5 Conclusions

This study examined the effects of crop rotation and monoculture systems on the quantity and quality of DOM components—dissolved organic carbon (DOC), nitrogen (DON), and phosphorus (DOP)—across three soil depths (0–30 cm, 30–60 cm, and 60–90 cm). Our findings establish soil depth as the primary determinant of DOM distribution, with concentrations decreasing and molecular complexity increasing with depth. Random Forest modeling identified total C, N, humus, and available P as key predictors of DOM variability, providing a framework for targeted soil management. The differential drivers of DOC, DON, and DOP reflect their distinct origins and regulatory pathways. These findings underscore the need for depth-specific strategies in soil carbon and nutrient management, especially in subsoils that act as long-term reservoirs. Sustainable practices like crop rotation that enhance organic matter accumulation and nutrient availability can improve DOM stability, support crop productivity, and contribute to climate resilience.

## Data Availability

The raw data supporting the conclusions of this article will be made available by the authors, without undue reservation.
